# Yap1 promotes the survival and self-renewal of breast tumor initiating cells via inhibiting Smad3 signaling

**DOI:** 10.18632/oncotarget.6655

**Published:** 2015-12-18

**Authors:** Jian-Guo Sun, Xie-Wan Chen, Lu-Ping Zhang, Jiang Wang, Max Diehn

**Affiliations:** ^1^ Cancer Institute, Stanford University School of Medicine, Stanford, California, USA; ^2^ Department of Oncology, Second Affiliated Hospital of Third Military Medical University, Chongqing, P.R. China

**Keywords:** Yap1, breast cancer, tumor initialing cells, self-renewal, Smad3

## Abstract

Tumor initiating cells (TICs) serve as the root of tumor growth. After identifying TICs in spontaneous breast tumors of the MMTV-Wnt1 mouse model, we confirmed the specific expression and activation of Yes-associated protein 1 (Yap1) within TICs. To investigate the role of Yap1 in the self-renewal of breast TICs and the underlying mechanism, we sorted CD49f^high^EpCAM^low^ cells as breast TICs. Active Yap1 with ectopic expression in breast TICs promoted their colony formation *in vitro* (*p*< 0.01) and self-renewal *in vivo* (*p*< 0.01), and led to a 4-fold increase in TIC frequency (*p*< 0.05). A conditional knock-out mouse was reconstructed to generate Yap1 knock-out breast tumors. The loss of Yap1 led to a dramatic growth disadvantage of breast TICs *in vitro* (*p*< 0.01) and *in vivo* (*p*< 0.01), and it also led to an over 200-fold decrease in TIC frequency (*p*< 0.01). The expression of active Yap1 was negatively correlated with that of phosphorylated Smad3 (p-Smad3). Transforming growth factor β (TGF-β) served as a strong enhancer of Smad3 and an inhibitor of clonogenesis of TICs. The presence of SIS3, a specific inhibitor of Smad3, could rescue the TGF-β -induced growth inhibition and reverse the Smad3 inhibition by Yap1. Analysis of a database containing 2,072 human breast cancer samples showed that higher expressions of Yap1 correlated with a poorer outcome of a 15-year survival rate and median overall survival (mOS)in patients, especially in those with basal breast tumors without estrogen receptor 1 (ER) expression. The findings indicate that active Yap1 promotes the self-renewal of breast TICs by inhibiting Smad3 signaling.

## INTRODUCTION

Breast cancer is the most common cancer among women and the leading cause of cancer-related death worldwide. Despite advances in molecular classification (luminal subtype, basal-like subtype, etc.) [1] and targeted molecular therapy (Herceptin, Lapatinib, etc.) [2, 3], breast cancer incidence (26 %) and mortality rate (15 %) still remain high [4]. Moreover, the mechanisms underlying breast cancer initiation and development are not fully understood.

Numerous studies suggest that tumor initiating cells (TICs), also called cancer stem cells (CSCs), are able to survive traditional chemotherapy and radiotherapy,as well as repopulate parental tumor cells after treatment [5]. By combining flow-cytometry (FACS) with serial xenograft transplantation, researchers have demonstrated the presence of TICs and their tumor initiating ability in multiple tumors including breast cancer [6]. Breast cancer usually contains two distinct populations, one of which express stemness markers and is identified as breast TICs [7]. Targeting these TICs holds the promise of eliminating the “root” of tumor cells, thus reducing relapse and metastasis [8]. However, understanding of the regulatory mechanisms that controls TIC self-renewal is still far from complete.

Yes-associated protein (Yap1) is a downstream effector of the Hippo pathway, and by controlling proliferation, it is a master regulator of organ size,normal tissue homeostasis, differentiation and apoptosis of normal stem cells [9, 10, 11]. Yap1 has been demonstrated to promote tumor growth, progression and metastasis in many solid tumors [12, 13]. Yap1 was also reported to be a new regulator of TICs or stem-like cancer cells in lung tumor [14] and esophageal cancer [15]. Previous research indicated that Yap1 was a potent oncogene that promotes breast cancer cells to metastasize *in vivo* to and proliferate in stiff surfaces *in vitro* [16, 17]. However, another study found that Yap1 acted as a tumor suppressor in breast cancer [18]. These controversial results may be largely due to the genetic variation within different cell lines used in individual studies. We hypothesized that Yap1 activation was critical in maintaining and regulating self-renewal of breast TICs. We therefore tested the function of Yap1 directly in primary breast tumors in a mouse model using gain-of-function and loss-of-function assays. Here we report that Yap1 is required for the survival and self-renewal of breast TICs via inhibiting Smad3 signaling.

## RESULTS

### Yap1 was expressed and activated within TICs

**Figure 1:**
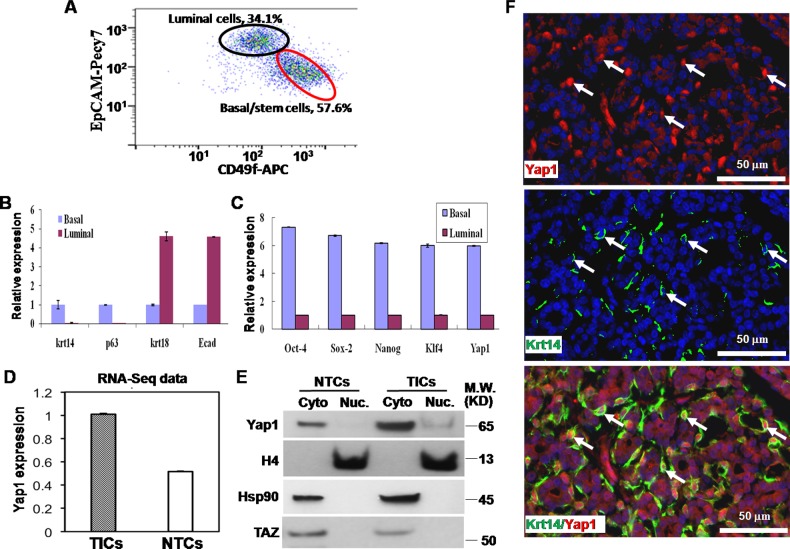
Yap1 is activated within tumor initiating cells (TICs) **A.** In FACS, two cell surface markers (EpCAM-Pecy7 and CD49f-APC) used to identify basal/stem cells (TICs, CD49f^high^EpCAM^low^, 57.6%) and luminal cells (NTCs, CD49f^low^EpCAM^high^, 34.1%) in primary breast tumor. **B.** Quantitative PCR showed that Krt14 and p63 were highly expressed in basal/stem cells, while krt18 and E-cadherin (Ecad) were highly expressed in luminal cells. **C.** Quantitative PCR showed a higher expression of Yap1 and embryonic stem factors (Oct-4, Nanog, Sox2, and Klf4) in basal/stem cells than in luminal cells. **D.** RNA sequencing (RNA-seq) showed a higher expression of Yap1 mRNA in TICs than in NTCs. **E.** Western blotting showed a significantly higher expression of Yap1 protein in TICs than in NTCs. Nuclear (Nuc.) Yap1 (i.e., active YAP1) was only detected in TICs while NTCs only contain cytoplasmic (Cyto) Yap1. There was no increase of TAZ in TICs compared with NTCs. Histone 4 (H4) and Hsp90 proteins were used as internal controls to show similar loading of Nuc and Cyto proteins, respectively. **F.** Dual-color immunofluorescence staining with TIC marker (Krt14, green) and Yap1 (red) antibodies. Although cytoplasmic Yap1 was detected in all tumor cells, strong nuclear expression of Yap1 was detected specifically in Krt14-positive TICs.

Previous research has identified TICs for primary breast tumors that spontaneously arose from MMTV-Wnt1 female mice, an animal model of human breast tumors [19, 20]. By adapting methods in a published report by our institute [19], we harvested and dissociated a tumor into a single cell suspension, and then cultured these cells *in vitro*. Two cell surface markers (EpCAM-Pecy7 and CD49f-APC) were used to identify 2 cell subsets using FACS, namely, basal/stem cells (TICs, CD49f^high^EpCAM^low^, approximately 57.6 %) and luminal cells (NTCs, CD49f^low^EpCAM^high^, approximately 34.1 %) in primary breast tumor (Figure 1A), as reported in a previous study [19]. CD49f^high^EpCAM^low^ cells were confirmed for their differentiation ability by expressing the genes found in mammary basal stem/progenitor cells, including p63 and Krt14. Additionally, CD49f^high^EpCAM^low^ cells were tested for tumor initiating ability with approximately 300 times NTCs in TIC frequency [19].

To verify the differentiation and self-renewal potential of TICs, we performed 2 experiments. First, similar to a prior report [30], we found that cell markers Krt14 and p63 were highly expressed in basal/stem cells and lowly expressed in luminal cells, while krt18 and E-cadherin (Ecad) were highly expressed in luminal cells and lowly expressed in basal/stem cells (Figure 1B). Second, TICs of any tumor highly express embryonic stem factors, such as Oct-4, Nanog, Sox2, and Klf4 [31]. Our experiments confirmed that these factors were more highly expressed in basal/stem cells than in luminal cells (Figure 1C). These results indicate that we have successfully isolated these 2 groups of cells, namely, TICs and NTCs.

Previously, our institute also analyzed the transcriptome profile of these TICs and NTCs using transcriptome sequencing (RNA-seq, GSE41286) [19]. Interestingly, we found that Yap1 mRNA was highly expressed in TICscompared with NTCs (Figure 1D). Here, we confirmed the Yap1 expression pattern using quantitative real-time PCR (qRT-PCR) on sorted TICs and NTCs. Yap1 mRNA expression was 5.98 times higher in TICs than in NTCs (Figure 1C). Next, consistent with mRNA expression level, Yap1 protein had a significantly higher expression in TICs. More importantly, Yap1 protein was only expressed in the cytoplasm of NTCs. However, Yap1 protein was more highly expressed in both the cytoplasm and the nucleus of TICs compared to those of NTCs (Figure 1E). The translocation of Yap1 into nucleus in TICs indicates the presence of active Yap1 within these cells.

Previously, studies found that invasive lobular carcinoma was characterized by a high expression of nuclear Yap1 [16]. To confirm this result, we performed dual-color immunofluorescence staining with a TIC marker (Krt14) antibody and a Yap1 antibody. Although cytoplasmic Yap1 could be detected in all tumor cells, strong nuclear expression of Yap1 was detected specifically in Krt14-positive TICs (Figure 1F). Taken together, Yap1 was highly expressed and specifically activated in TICs, suggesting its potential function within these cells.

### Ectopic active Yap1 increased the clonogenesis of breast TICs

**Figure 2:**
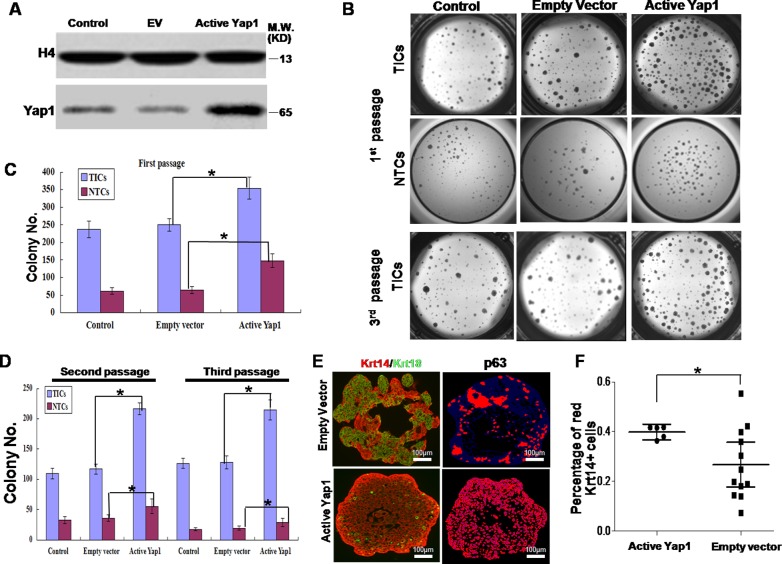
Ectopic active Yap1 increased clonogenesis of breast TICs **A.** After lentivirus transfection, western blotting showed a more active form of Yap1 in the nucleus in lentivirus-Yap1 transfected TICs than in parental TICs and empty vector (EV) transfected TICs. **B.** 1^st^ passage of breast TICs and NTCs harboring active Yap1 generated more colonies in 3-dimentional (3D) culture system. 3^rd^ passage of breast TICs harboring ectopic active Yap1 yielded more colonies. **C.** Colony number count of TICs and NTCs in the 1^st^ passage from **B.** (*, *p* < 0.001, *n* = 8). **D.** Colony number count of TICs and NTCs in the 2^nd^ and 3^rd^ passage from **B.** (*, *p* < 0.001, *n* = 6). **E.** Immunostaining analysis of colonies formed by TICs with or without ectopic expression of active Yap1. TIC markers (p63 and Krt14) and NTC markers (Krt18). Laser confocal microscopy. **F.** Image histogram analysis of images from **E.**. Percentage of red Krt14+ cells was indicated (*, *p* < 0.01, *n* = 15).

To test the function of Yap1 in breast TICs, we introduced active Yap1 with a S127A mutation into TICs using lentivirus infection (lentivirus-Yap1). Next, qRT-PCR (Supplementary Figure S1C) and western blotting (Figure 2A) showed more active Yap1 in the nucleus of TICs transfected with lentivirus-Yap1 than inthe nucleus of those transfected with an empty vector (EV). As shown in Figure 2B and 2C, the TICs harboringactive Yap1 generated more colonies, indicating the expansion of these TICs *in vitro* (*p*< 0.001, n = 8). Similarly, NTCs could form more colonies in the presence of active Yap1 (*p*< 0.001, n = 8, Figure 2B and 2C).

Expansion *in vitro* could involve self-renewal or proliferation and differentiation into mature cell types. To test whether Yap1 activation was correlated with TIC self-renewal, we utilized several established methods [25]. Serial passage *in vitro* was established previously to evaluate the self-renewal abilities of TICs [32]. In the current study, we dissociated derived colonies from lentivirus infected TICs, sorted GFP^+^ cells (introduced by successful lentivirus infection) and passaged these cells at least 3 times *in vitro*. For the same number of seeding cells, TICs with active Yap1 consistently had more colonies than TICs with EV transfection (*p*< 0.001, n= 6, Figure 2B and 2D), suggesting that active Yap1 could promote TIC self-renewal. In addition, NTCs formed much fewer colonies at the second passage and third passage than TICs; however increasing colonies formed with active Yap1 (*p*< 0.001, n= 6, Figure 2D).

Next, we examined the differentiation status of these TIC colonies using active Yap1. By analyzing TIC colonies with dual-color immunofluorescence, we detected both TIC markers (p63 and Krt14) and a NTC marker (Krt18). The findings suggested that TICs could both self-renew and differentiate into NTCs in this 3D culture system, which was consistent with previous reports [19]. After introduction of active Yap1, TIC colonies contained significantly more p63- and Krt14-positive cells, but fewer Krt18-positive cells (Figure 2E and 2F), indicating that active Yap1 promotes self-renewal and inhibits differentiation of TICs.

### Ectopic active Yap1 increased breast TIC frequency *in vivo*

**Figure 3:**
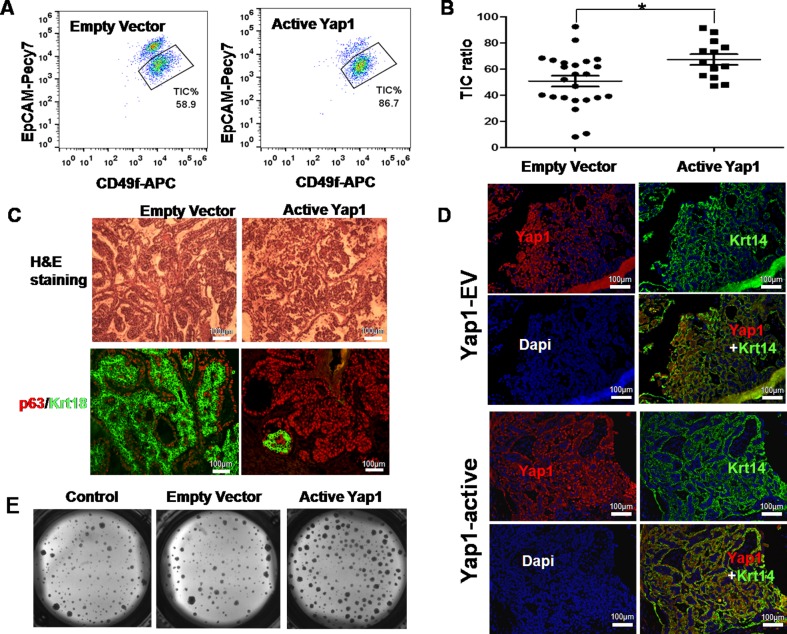
Ectopic active Yap1 increased breast TIC frequency *in vivo* **A.** A FACS profile revealed a significant increase of TICs in Yap1 active tumors (86.7%) compared with empty vector transfected tumors (58.9%). **B.** Quantification of TIC ratio from **A.** (*, *p* = 0.0079, *n* = 13). **C.** Histologic analysis of mammary tumors with/without ectopic expression of active Yap1. Hematoxylin and Eosin (H&E) staining and immunostaining with TIC-marker (p63) and NTC marker (Krt18). **D.** In a transplanted tumor infected with active Yap1, confocal microscopy showed that a larger number of active Yap1 expression was co-localized with Krt14-positive cells. **E.** Colonies formed by breast TICs sorted from mammary tumors in **A.** with/without ectopic expression of active Yap1 (*p* < 0.001, *n* = 6).

To investigate whether active Yap1 promotes self-renewal and inhibits the differentiation of TIC *in vivo*, we dissociated resulting tumors into a single cell suspension and analyzed cell fractions using FACS. Our FACS profile also revealed a significant increase of TICs in Yap1 active tumors (n= 13, TIC % = 86.7 %) compared with empty vector transfected tumors (n= 24, TIC % = 58.9 %) (*p* = 0.0079, Figure 3A and 3B).

We also transfected TICs with lentivirus-Yap1 or -EV, sorted infected TICs (GFP^+^), and then transplanted them to syngeneic mice. Transplantation could result in tumor growth in both groups. The resulted tumors displayed distinct morphologies (Figure 3C). As indicated by H&E staining, Yap1 active tumors showed no distinct structures compared with EV controls, and they contained more TIC marker p63-positive cells and fewer NTC marker Krt18-positive cells. This result suggests that active Yap1 promotes self-renewal and inhibits differentiation of TICs *in vivo*. Moreover, confocal microscopy showed that a large number of active Yap1 expression was co-localized with Krt14-positive cells, which confirmed the successful infection of lentivirus transducing active Yap1 (Figure 3D). Compared with EV control, Yap1 expression in the nucleus of TICs from Yap1 active tumors was dramatically higher (Figure 3D). Nevertheless, with the inoculation of the same number of TICs with or without active Yap1 in FVB/NJ female mice, active Yap1 formed dramatically bigger tumors than mice with the EV control (Supplementary Figure S1D). We then sorted out TICs and tested their colony forming ability *in vitro*. Consistent with *in vivo* data, Yap1 active TICs gave rise to much more colonies than TICs infected with empty vectors (*p*< 0.001, n = 6, Figure 3E).

Lastly, we tested whether the increase of the TIC fraction was consistent with tumor initiating ability *in vivo* in Yap1 active tumors. For this aim, we sorted GFP^+^ tumor cells and performed a limited dilution assay (LDA) by transplanting these cells into syngeneic mice, and then we examined possible tumor growth. As a result, we found at least one tumor initiating event (tumor growth) in 811 TICs transfected with empty vectors (TIC frequency of 1/811) and 173 TICs with active Yap1 (TIC frequency of 1/173). By comparison, the tumor initiating events in tumors with active Yap1 were 4 times higher (*p*< 0.01, Table 1). Taken together, active Yap1 could promote TIC self-renewal and increase TIC frequency in breast tumors.

**Table 1:** *In vivo* LDA of breast tumor cells in active Yap1- or Yap1-ko treated cells

Treatment groups	Number of cells injected	Number of tumor formation/total number of injections	TIC frequency (95 % CI)
Empty Vector	2,000	7/8	1/789 (1/462-1/1349)
	1,000	5/8	
	400	4/8	
	80	2/8	
Active Yap1	2,000	3/4	1/251^*^ (1/129-1/488)
	1,000	4/4	
	400	8/8	
	80	6/8	
Yap1-flp	100,000	8/8	1/715 (1/252-1/2025)
	1,000	3/4	
	200	1/4	
Yap1-ko	100,000	1/4	1/174,779^**^ (1/39273-777833)
	1,000	1/4	
	200	0/4	

* *p* < 0.01, compared with Empty vector;

** *p* < 0.001, compared with Yap1-flp.

### Yap1-ko inhibited the growth of breast TICs *in vitro* and *in vivo*

**Figure 4:**
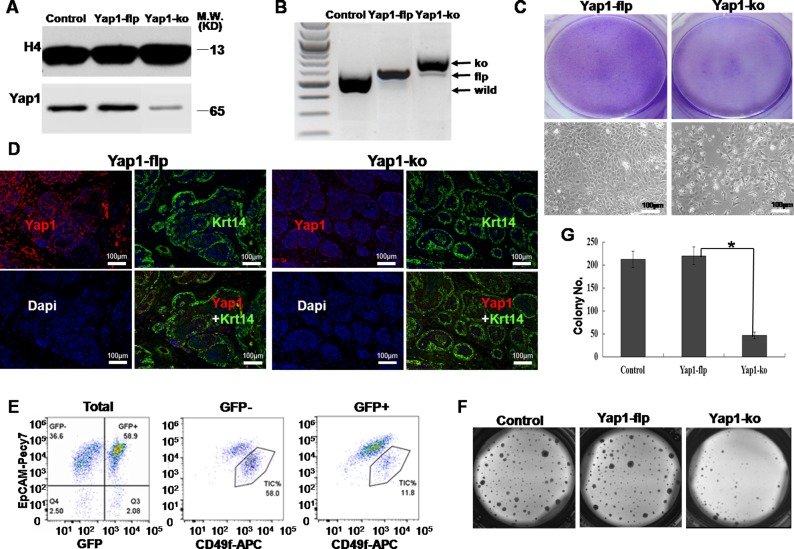
Yap1-ko inhibited the growth of breast TICs *in vitro* and *in vivo* **A.** Western blotting showed a less active form of Yap1 in the nucleus of Yap1-ko TICs than in parental TICs and Yap1-flp TICs. **B.** Genotyping of genomic DNA showed that the Yap1 gene was successfully knocked out of Yap1-ko mice with tamoxifen injection compared with Yap1-flp mice. **C.** In 2D culture, Giemsa staining and bright field imaging indicated a cell density originating from breast TICs sorted from mammary tumor with/without endogenous Yap1 deletion (Yap1-ko). **D.** In transplanted tumor withYap1-ko, confocal microscopy showed that less signal of active Yap1 was co-localized with Krt14-positive cells. **E.** Flow cytometry analysis of TIC fractions in mammary tumors after tamoxifen injection. TIC fractions were analyzed separately for GFP+ cells with Yap1 KO (Yap1-ko) and GFP- cells with functional endogenous Yap1 (Yap1-flp). **F.** Colonies formed by breast TICs sorted from mammary tumor in **E.** with/without Yap1 deletion (Yap1-ko). **G.** Relative cell numbers indicated cell growth in **F.** for mammary tumor cells with/without Yap1-ko (*, *p* = 0.006, *n* = 6).

Next, we asked whether knock-out of Yap1 (Yap1-ko) could specifically block TIC growth both *in vivo* and *in vitro*. After several generations of breeding, the MMTV-Wnt1/Rosa26-Cre/mTmG/Yap1^flox/flox^ (Yap1-flp) mice were obtained and confirmed by genotyping (Supplementary Figure S1B). The resulting female mice also gave rise to breast tumors, as did the original MMTV-Wnt1 single transgenic mice. To knockout Yap1 (Yap1-ko) in breast TICs, which were sorted from Yap1-flp breast tumors *in vitro*, we infected these cells with adenovirus-Cre. A Yap1 decrease in Yap1-ko TICs was successfully confirmed at the mRNA level using qRT-PCR (Supplementary Figure S1E) and at protein level in the nucleus using western blotting (Figure 4A).

To study Yap1 function within these tumors *in vivo*, we continuously injected tamoxifen into mice bearing breast tumors for 2 weeks, and then we waited for another 3 weeks to harvest these tumors. Genotyping of genomic DNA showed Yap1-ko with tamoxifen injection (Figure 4B) that was consistent with previous research [24].

We tested the effect of Yap-ko *in vitro* and *in vivo*. First, compared with the control, Yap1-ko significantly reduced the cell density and number that originated from the same number of seeding cells in the 2D collagen culture (*p*< 0.001, n = 6, Figure 4C). Second, confocal microscopy showed that Yap1-positive cells were dramatically downregulated under the condition of tamoxifen injection and were not co-localized with Krt14-positive cells, which confirmed the successful knock-out of Yap1 (Figure 4D). Compared with TICs from the Yap1-flp tumor, Yap1 expression in the nucleus of TICs from Yap1-ko tumors was dramatically lower (Figure 4D). FACS analysis of these tumors revealed that approximately 60 % of GFP^+^ cells switched from original tdTomato^+^GFP^−^ cells, indicating active Cre recombinase and knock-out of Yap1 within these cells (Figure 4E). Compared with the remaining 40 % tdTomato^+^GFP^−^ cells without Cre recombination, GFP^+^ cells contained approximately 5 times fewer TICs (58.9 % vs. 11.8 %), suggesting a growth disadvantage for TICs with Yap1-ko *in vivo* (Figure 4E).

To confirm this result *in vitro*, we sorted TICs for both GFP^+^ and GFP^−^ tumor cells. Compared with GFP^−^ control cells (Yap1-flp), GFP^+^ cells (Yap1-ko) showed a significantly smaller colony number, suggesting that Yap1 was required by TICs to grow in a 3D matrigel culture system (*p*= 0.006, n = 6, Figure 4F and 4G).

Finally, to directly test whether a Yap1-ko could inhibit tumor initiating ability within breast tumors, we performed LDA *in vivo*. As a result, we estimated that there was at least one tumor initiating event from 714 Yap1-flp tumor cells (TIC frequency of 1/714). However, Yap1-ko led to a more than 200 times lower TIC frequency (1/174,779) (*p*< 0.001, Table 1). With the inoculation of the same number of TICs with Yap1-flp or Yap1-ko in FVB/NJ female mice, Yap1-ko formed dramatically smaller tumors compared to mice with Yap1-flp TICs (Supplementary Figure S1D). Collectively these results demonstrate that Yap1 activation was required for the expansion of breast tumor cells and the self-renewal of breast TICs *in vitro* and *in vivo*.

### Yap1 promoted the self-renewal of breast TICs via inhibiting Smad3 signaling

**Figure 5:**
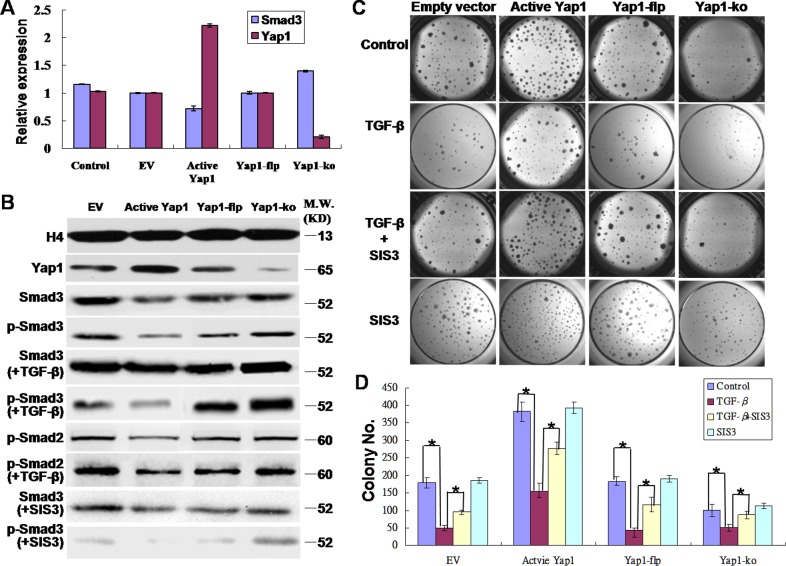
Yap1 promotes the self-renewal of breast TICs via inhibiting Smad3 **A.** Real-time PCR revealed that Yap1 inhibited Smad3 signaling. The change in Smad3 mRNA expression was opposite to that of Yap1 mRNA expression in both the active Yap1 group and the Yap1-ko group. **B.** Western blotting demonstrated that Yap1 inhibited phospho-Smad3 (p-Smad3) signaling. TGF-β enhanced p-Smad2/3 expression whereas SIS3 inhibited p-Smad3 expression (blots spliced). **C.** Colony formation with or without ectopic active Yap1 introduced by lentivirus infection, with or without Yap1-ko induced by tamoxifen injection, and with or without TGF-β and SIS3 intervention. **D.** Quantification of colony formation from **C.** (*, *p* < 0.001, *n* = 6).

In the current study, both qRT-PCR and western blotting showed that the change of Smad3 expressions was opposite to that of Yap1 expression in both the active Yap1 group and the Yap1-ko group at both mRNA (Figure 5A) and protein levels (Figure 5B). Thus, the expression of Yap1 was negatively correlated with total Smad3 expression. Furthermore, the detection of phosphorylated Smad3 (p-Smad3), the active form of functional Smad3, also showed a negative correlation with the expression of Yap1, which suggested that Yap1 inhibited the expression of both total Smad3 and p-Smad3. Smad3 is downstream of the transforming growth factor β (TGF-β) signaling pathway, and the TGF-β/Smad3 signaling pathway is a potent growth inhibitor for most cancer types [33]. In our study, 100 pg/mL of TGF-β served as a strong enhancer of Smad3, and the presence of 1-μM SIS3 (Calbiochem, #566405) for 30 min, a specific inhibitor of Smad3 [34], blocked the protein expression of p-Smad3 (Figure 5B). TGF-β signals through the closely-related Smad2 and Smad3 proteins, among others. Next, we evaluated the expression of p-Smad2 in TGF-β induction. Western blotting demonstrated that TGF-β also enhanced the expression of p-Smad2 in both the active Yap1 group and the Yap1-ko group (Figure 5B).

In addition, we tested the roles of TGF-β and SIS3 in affecting clonogenesis of breast TICs in 4 groups (i.e., EV, active Yap1, Yap1-flp and Yap1-ko). Consistent with the above experiments, active Yap1 increased colony numbers, and Yap1-ko significantly decreased colony numbers (*p*< 0.001, n = 6, Figure 5C and 5D). TGF-β was a strong inhibitor of clonogenesis of breast TICs, and SIS3 rescued the TGF-β-induced growth inhibition in these 4 groups (*p*< 0.001, n = 6, Figure 5C and 5D), while SIS3 alone did not change the colony numbers compared with that of the control (Figure 5C and 5D). All of these results indicated that active Yap1 promoted the self-renewal of breast TICs by inhibiting Smad3 signaling.

### Yap1 was correlated with the outcomes of human breast tumor patients

**Figure 6:**
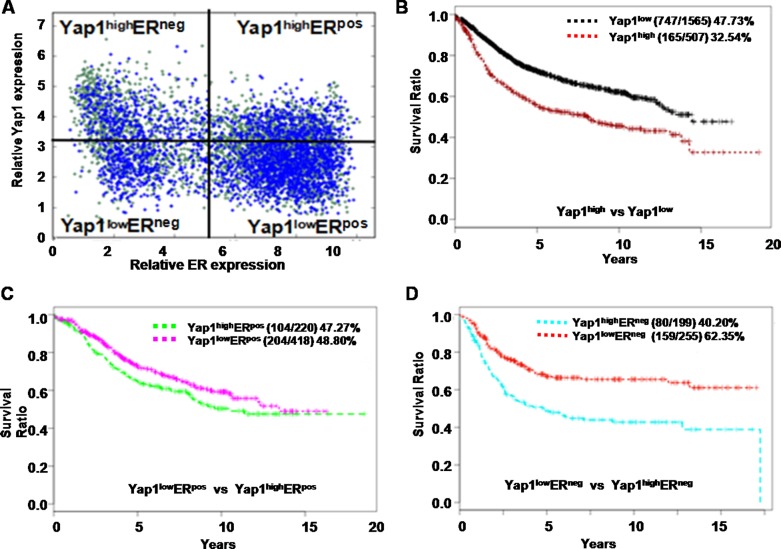
Yap1 is correlated with the outcome of human breast cancer patients **A.** Based on expression levels of the estrogen receptor (ER) and the Yap1 gene, 2, 072 human breast tumor samples were divided into 4 sections for subsequent survival analysis. **B.** The 15-year survival rate of the Yap1^high^ group (165/507) was more closely correlated with a poor prognosis than was that of the Yap1^low^ group (747 / 1,565) (*p* < 0.05). **C.** In ER-positive cancers (ER^pos^), the Yap1^high^ group did not correlate with patient survival outcome compared with the Yap1^low^ group (104/220 *vs.* 204/418, *p* > 0.05) **D.** In ER-negative cancers (ER^neg^), the Yap1^high^ group was correlated with a lower survival rate in patients than the Yap1^low^ (80/199 *vs.* 159/255, *p* < 0.05).

The Gene pyramid database we used in current study contained 2,072 human breast cancer samples that had both gene expression profiles and patient survival data (Figure 6A). Solely based on the Yap1 expression level, the 15-year survival rate of Yap1^high^ status (165 survived patients / 507 start total patients) was more strongly associated with a poor prognosis compared to Yap1^low^ (747 survived patients / 1565 start total patients) (*p*< 0.05, Figure 6B).

However, this result could also be misleading because the analysis did not differentiate breast cancer subtypes (such as basal cancers and luminal cancers), which could lead to distinct patient outcomes. As widely known, there is a significant correlation between histological types and molecular subgroups, and luminal breast cancers usually express the ER and have a better prognosis than basal breast cancers [1, 35]. Therefore, we further utilized the ER status to differentiate these 2 breast cancer subtypes, and we examined the correlation of Yap1 expression in each of these cancer types. Interestingly, within ER-positive cancers (ER^pos^), the Yap1^high^ status did not correlate with patient survival outcome compared with the Yap1^low^ status (104/220 vs. 204/418, *p*> 0.05) (Figure 6C, Table 2). However, in ER-negative cancers (ER^neg^), the Yap1^high^ status consistently had a lower survival rate in human patients compared with the Yap1^low^ status (80/199 vs. 159/255, *p<* 0.05) (Figure 6D, Table 2), suggesting that Yap1 may be a potential driver gene for treating this breast cancer subtype. We also analyzed the median overall survival (mOS) of different groups. As a result, Yap1 status was an independent poor prognosis factor of mOS in breast cancer that was consistent with the 15-year survival rate (7.8 yr. vs. 13.9 yr. in Yap1^high^ and Yap1^low^, *p*< 0.05). Moreover, Yap1 expression was also associated with cancer type and ER expression in mOS evaluation. There was no significant difference in ER^pos^ patients between Yap1^high^ (10.3 years) and Yap1^low^ groups (13.0 years) (*p*> 0.05), while there was a significant difference in ER^neg^ patients between Yap1^high^ (6.1 years) and Yap1^low^ groups (no reach, NR) (*p<* 0.05) (Table 2).

**Table 2:** Yap1 indicates poor prognosis of breast cancer patients with an ER- negative status

Yap1/ER status	Yap1 expression		ER-positive		ER-negative	
	high	low	Yap1 high	Yap1 low	Yap1 high	Yap1 low
Start pts	507	1565	220	418	199	255
Survived pts	165	747	104	204	80	159
15-yr survival rate (%)	32.54	47.73	47.27	48.80	40.20	62.35
*p* value	0.0000		0.0670		0.0000	
Median OS (yr)	7.8	13.9	10.3	13.0	6.1	NR
*p* value	*p* < 0.05		*p* > 0.05		*p* < 0.05	

## DISCUSSION

Despite the fact that Yap1 has been examined in normal stem cells from several tissues, its function in breast stem cells or breast TICs has not been directly tested. In the present study, starting with high-throughput RNA-seq data, we identified Yap1 by its specific expression and activation within breast tumor TICs. Using a MMTV-Wnt1 mouse model of breast tumor, further functional assays implicate a critical role for Yap1 in regulating the self-renewal of TICs within this mouse tumor. To our knowledge, we are the first to test Yap1 function in TICs from primary breast tumor instead of from a cell line, as the former can more closely reflect the true features of the TICs from human breast tumors.

Because basic expression of active Yap1 is present in TICs, our lentivirus vector further enhanced active Yap1 and dramatically promoted TIC self-renewal and tumor initiation in serial passages. Interestingly, active Yap1 in NTCs enhanced clonogenesis of NTCs in the 3D culture only at the first passage. Thus, we can conclude that active Yap1 promotes the self-renewal and tumor initiation of TICs but not NTCs.

Next, we asked whether Yap1 activation was required for breast tumor cell growth and self-renewal of breast TICs. Consequently, the loss of Yap1 led to a dramatic growth disadvantage of TICs both *in vivo* and *in vitro*, and it significantly decreased TIC frequency within these breast tumors. Additionally, the only two tumor growths originating from Yap1-ko cells contained approximately 80 % GFP^−^tdTomato^+^ cells harboring a functional Yap1 gene. This contamination of GFP^−^ cells may originate from FACS sorting whose purity was approximately 98 %. However, this original contamination of ∼2 % GFP^−^ cells expanded to final ∼80 % GFP^−^ cells within resulting tumors over one month of growth *in vivo*, suggesting a greater growth advantage of tumor cells with intact Yap1 than cells with Yap1 deletion. Therefore, the actual TIC frequency within Yap1-ko tumor cells should be even lower than we detected here. Taken together, Yap1 was required by breast TICs to survive and expand both *in vivo* and *in vitro*. These data implicate a critical role for Yap1 in promoting the self-renewal of breast TICs. Our findings here strengthen previous reports that Yap1 endows esophageal cancer cells with stem-like properties [15] and link tumor progression with lung tumor propagating cells (TPCs) [14].

TAZ (a paralog of Yap1) as a downstream effector of the Hippo pathway is highly expressed in many human cancers [36]. TAZ is critical for maintaining normal basal/stem cells in normal breast tissues [37]. TAZ has also been demonstrated to be essential for promoting the expansion of breast TICs within cell lines [38, 39]. Interestingly, our RNA-seq analysis indicated that TAZ was not highly expressed in TICs from primary mammary tumors at both mRNA and protein levels, while Yap1 was specifically activated within these cells (Supplementary Figure S2A, Figure 1E). It is possible that different breast tumors could depend on the hippo pathway via different effectors. Further studies are needed to distinguish the function of these two effectors in primary breast cancer.

As a transcriptional coactivator, Yap1 could upregulate or downregulate downstream genes in different tumor types or in different phases of tumor development. Yap1 directly upregulated SOX9, and served as a primary determinant of TIC properties in non-transformed cells and in esophageal cancer cells [15]. A recent study also reported that β-Catenin-driven cancer cells required Yap1 to survive [40, 41]. The β-Catenin-Yap1-TBX5 complex is critical for the survival of β-Catenin-driven cancers, such as colon cancer and other cancers [40]. Moreover, several studies have reported that TAZ shared similar target genes with Yap1, such as β-Catenin, TGF-β, Smad and CTGF [42, 43, 44]. In the present study, we tested these genes and their correlation with Yap1. However, we did not find that significant changes in the expression of these genes correlated with Yap1 activation or deletion (data not shown), with the exception of Smad3. Because our study is the first to focus on the MMTV-Wnt1 breast tumor, the result may be different from previous studies [38, 39]. In our experiment,we found that Yap1 was negatively correlated with p-Smad3 expression. Moreover, TGF-β was used as a strong enhancer of p-Smad3 and a strong inhibitor of clonogenesis of breast TICs. Furthermore, SIS3 rescued the growth inhibition induced by TGF-β. Our results indicate that Yap1 promotes the self-renewal of breast TICs by inhibiting Smad3. Based on the experiments demonstrating that Yap1 promotes the self-renewal of TICs, we could conclude that elevated Yap1 probably compensates for the role of TAZ in breast TICs.

Chen CL et al. [45] reported that Yap1 was responsible for the cytoplasmic retention of Smad3 and that it inhibited Smad3 in hepatocellular carcinoma (HCC). Moreover, Yap1 activates the TGF-β-induced epithelial-to-mesenchymal transition (EMT) in non-transformed mammary epithelial cells [46] and initiates the development of the heart valve [47]. Taken together, these observations show that Yap1 differently regulates the TGF-β/Smad signaling pathway in the development of different tissues. It is well-known that TGF-β inhibits tumor growth in the early stage of tumorigenesis [33] and that it promotes metastasis by inducing EMT in advanced tumors [48]. Thus, Yap1 appears to promote tumor development by not only blocking growth inhibition but also by activating the EMT that is induced by the TGF-β/Smad signaling pathway.

TGF-β/Smad induced ROS production in hepatocellular carcinoma cells [49]. Moreover, ROS is critical for regulating the self-renewal of stem cells and tumor TICs in several tissues, including breast tissue [50, 51]. The ROS level in cancer cells is associated with the inhibition of tumorigenesis and tumor growth [52]. The key gene that regulates ROS levels within cells is still unknown. Yap1, on the other hand, is crucial for maintaining mitochondrial activities and the cellular ROS level [53]. Therefore, it is possible that Yap1 could promote TIC self-renewal by reducing cellular ROS levels. In our study, cytoplasmic ROS was measured using CellROX™ Deep Red. Intriguingly, our preliminary data confirmed that ectopic expression of active Yap1 dramatically decreased the cytoplasmic ROS level in breast TICs (Supplementary Figure S2B and S2C), while Yap1-ko increased ROS stresses in breast TICs (Supplementary Figure S2B and S2D). However, Tempo, a ROS reducing drug [54], did not display dramatic rescuing effects on TICs with Yap1-ko by forming colonies compared with the control (data not shown). This result suggests that Yap1 may promote TIC self-renewal through multiple pathways bypassing ROS pathways.

Additionally, it was reported that Yap1 overexpression was associated with the lymph node metastasis, poor prognosis and progression of colorectal cancer [55, 56], gastric carcinoma [57] and ovarian cancer [58]. It was also reported that TIC expansion and progression were usually correlated with a poor patient outcome, that is, the more aggressive the TICs, the higher the risk for breast cancer relapse and metastasis, which would thus lead to poor prognosis. Indeed, the TIC frequency is correlated with the invasive potential of breast cancer cells [59]. Here we asked whether the regulatory effect of Yap1 on TICs could be extended to human breast cancer and predict prognosis in basal breast tumors. We found that Yap1 could independently predict prognosis in 2,072 breast tumor patients. Notably, Yap1 expression was selectively correlated with survival outcomes in basal breast tumor patients. Nevertheless, a study showed that a decrease in Yap1 expression was an independent prognostic factor for recurrence in a subgroup with a less aggressive luminal A breast cancer, which may possibly be explained by the decreased tamoxifen sensitivity that is induced by Yap1 downregulation [60]. All of these studies suggest that Yap1 functions in breast cancer initiation and treatment sensitivity are complex.

Taken together, our results suggest that Yap1 is sufficient to promote TIC self-renewal and is required for TIC maintenance. Yap1 is important in breast tumor growth, progression and metastasis, and could be a potential therapeutic target for specifically eliminating breast TICs, thus greatly improving the prognosis of human breast tumors.

Using a primary mouse breast tumor model, we show that ectopic expression of active Yap1 increases TIC self-renewal and TIC frequency within these tumors, while the loss of endogenous Yap1 disrupts TIC self-renewal and decreases TIC frequency. Moreover, active Yap1 promotes the self-renewal of breast TICs by inhibiting Smad3 signaling. We further demonstrate that our data are consistent with the survival data from human breast cancer patients, showing that elevated Yap1 expression is correlated with a poor survival outcome, especially for basal-type breast cancers. Therefore, our study confirms that Yap1 is a potential drug target for these human breast tumors.

## MATERIALS AND METHODS

### Transgenic mice, tumor dissociation and FACS

MMTV-Wnt1 mice were purchased from the Jackson laboratory (Jackson lab, #002934). MMTV-Wnt1 murine breast tumors were harvested and dissociated into single cell suspensions [19, 20]. The obtained cell suspensions were stained with antibodies and sorted with a FACS Aria II cell sorter (BD Biosciences). We used antibodies including anti-mouse CD49f-APC (eBiosence, #17-0495-82) and EpCAM-Pecy7 (Biolegend, #118206) for sorting TICs (CD49f^high^EpCAM^low^) and non-tumorigenic cells (NTCs, CD49f^low^EpCAM^high^). All of the animal procedures were conducted following animal regulations, guidelines and protocols approved by an Administrative Panel on Laboratory Animal Care (APLAC) at Stanford University.

### RNA sequencing (RNA-seq)

Methods for generating and analyzing the RNA-seq data are available in our previous study [19]. Sequencing data are accessible through Gene Expression Omnibus (GSE41286).

### Lentivirus infection

Lentiviruses were produced and purified as previously described [21]. Briefly, active Yap1 cDNA with a S127A mutation [22, 23] was cloned into the lentivirus vector pCDH (System biosciences, #CD521A-1). Lentivirus vectors were co-transfected with packaging plasmids into HEK-293T cells (ATCC, #CRL-11268) to produce lentivirus particles. Active viruses were mixed with sorted tumor cells supplemented with 8 μg/mL of Polybrene (Sigma, #H9268), and seeded to a 3D matrigel culture system. Successfully transduced cells were sorted using FACS based on GFP expression introduced by lentivirus vector. Generated biohazard wastes were treated with 10 % bleach to de-activate possible virus particles before dumping into sewage. All of the protocols meet the biosafety regulation policies of Stanford University.

### Conditional Yap1 knock-out mice

Yap1^flox/flox^ mouse was kindly provided by Dr. Pan [24] and mated to Rosa26-Cre mice (Jackson lab, #012429), mTmG mice (Jackson lab, #007576) and MMTV-Wnt1 mice (Jackson lab, #002934) to generate MMTV-Wnt1/Rosa26-Cre/mTmG/Yap1^flox/flox^ (Yap1-flp) mice. The generated female mice not only had MMTV-Wnt1 transgene but also a Rosa26-CreER2 transgene and a reporter transgenic mTmG. The mTmG reporter was used as an indicator of active Cre recombinase, which switched from red-fluorescence (tdTomato) to green-fluorescence (GFP) upon active Cre recombinase (Supplementary Figure S1A and S1B). Tamoxifen (Sigma, #T5648) was dissolved in corn oil (Sigma, C8267) to reach a concentration of 5 mg/mL and injected subcutaneously for 10 successive days to knock out the Yap1 gene *in vivo*. DNA extraction and genotyping from tail biopsies were performed as previously described [24]. Primers for genotyping of Yap1, Cre and Wnt (Supplementary Table S1) were used to identify MMTV-Wnt1/Rosa26-Cre/mTmG/Yap1^flox/flox^ mice. Standard genotyping protocol followed the instruction of previous research [24] and that of the Jackson Laboratory. Similarly, 1 μg/mL of 4-Hydroxytamoxifen (Sigma, #68047-06-3) was supplemented in culture medium to knock out the Yap1 gene *in vitro*. Alternatively, active adenovirus-Cre (Vector Biolabs, #1045) was used to knock out the Yap1^flox/flox^ allele (Yap1-flp) *in vitro*.

### Quantitative RT-PCR (qRT-PCR)

Quantitative RT-PCR was performed as previously described [25]. Briefly, total RNA was extracted from sorted cells using the RNeasy Micro Plus Kit (Qiagen, #74034) and reversely transcribed to cDNA using standard techniques (ABI). Quantitative PCR was performed using SYBR® Green PCR Master Mix (ABI, #4309155). All of the primers used in current study were designed by IDT (www.idtdna.com) and synthesized by Elim Biopharm (www.elimbio.com). The primer sequences are listed in Supplementary Table S1.

### Western blotting

Nuclear and cytoplasmic proteins were extracted according to the manufacturer's instructions (Sigma, #NXTRACT). Western blotting was performed as described in detail in a previous study [26]. Primary antibodies used in current study included anti-mouse Yap1 (Abcam, #ab56701), TAZ (Cell signaling, #4883), Histone H4 (Santa Cruz, #sc-25260), Hsp90 (Santa Cruz, #sc-8262), p-Smad2 (Santa Cruz, #sc-101801), Smad3 (Cell signaling, #9513), p-Smad3 (Santa Cruz, #sc-11769), Krt14 (Santa Cruz, #sc-43310), Krt18 (Santa Cruz, #sc-45406) and p63 (Abcam, #ab124762).

### Immunofluorescence

For immunofluorescence, tumor tissue was embedded in paraffin and cut into slices of 7 μm thickness for immunostaining. Immunostaining was carried out according to the manufacturer's instructions (Vector Lab, #BMK-2202). DAPI solution (1:1,000, Sigma, #28718-90-3) was used as a nuclear stain. DyLight 594 AffiniPure Goat Anti-Mouse IgG (1:1,000 dilution; Abbkine, #A23410) and DyLight 488 AffiniPure Goat Anti-Mouse IgG (1:1,000 dilution; Abbkine, #A23210) were used as secondary antibodies for red and green fluorescence, respectively. The images were taken using a laser confocal microscope.

### Two-Dimensional (2D) tumor growth assay in collagen gel

Collagen gel solution (Chemicon, #ECM675) was prepared according to the manufacturer's instructions (i.e., 8 mL of collagen solution, 2 mL of 5x medium and 250 μL of neutralization solution). After mixing, 1.5 mL of the chilled collagen solution was added onto a 6-well tissue culture plate. After the collagen gel was formed, 3 mL of culture medium containing cells was added into the plate. After 10-14 days, cells were stained by Giemsa solution (Sigma, #G9641) to observe the cell density and number.

### Three-Dimensional (3D) tumor spheroid assay in matrigel

FACS-sorted tumor cells were suspended in the culture medium consisting of DMEM/F12 (Invitrogen), 20 ng/mL of mouse EGF (BD, #354001), 20 ng/mL of human FGF (BD, #354060), 10 μM of Y-27632 (Sigma, #Y0503) and 1x B27 (Invitrogen, #17504-044), and they were plated on top of solidified matrigel (BD Bioscience, #356237). Then, 100 μL of the cell suspension with 4k cells was put onto the matrigel, and the plate was incubated at 37°C. After 10-14 days, colonieswere counted under an inverted light microscope. To passage, colonies were dissociated with 1 mg/mL of dispase (Invitrogen, #17105-041) and treated with trypsin/0.05 % EDTA (Gibco, #25200) to a produce single cell suspension [27].

### Evaluation of cellular ROS production

A total of 10,000 sorted cells were incubated with 5 μM of CellROX™ Deep Red (Invitrogen, #c10422), a fluorescent indicator of cytoplasmic ROS, in culture medium for 30 min. A total of 5 μg/mL of Hoechst-33342 (Sigma, #H6024) was then added as a nuclear stain. After being washed twice, cells were resuspended in HBSS with 2 % Calf serum and analyzed by flow cytometry.

### Limited dilution assay (LDA)

Double sorted cells by FACS were suspended in staining buffer, HBSS with 2 % calf serum (Gemcell, 100-506) and with 25 % matrigel (BD Bioscience, #356237), and then injected subcutaneously into the vicinity of the mammary fat pads in the syngeneic mouse (FVB/NJ female mice, aged 3–6 weeks). The mice were anesthetized by inhalation of isoflurane (Baxter Healthcare Corporation, #NDC10019-773-60). After injection, the mice were observed weekly for up to 6 months for tumor formation. Tumor initiating cell frequencies were calculated using ELDA (http://bioinf.wehi.edu.au) [28].

### Prognosis analysis of Yap1 expressionin breast tumor patients

Public databases were used to test the correlation between Yap1 expression and the outcomes of human breast tumor patients using Boolean analysis (http://genepyramid.stanford.edu/microarray/Explore/explore.php) [29]. Yap1 expression and ER status were analyzed. The 15-year survival rate and mOS were calculated and compared under different conditions.

### Statistical analysis

Replicate numbers represent biological replicates using distinct tumors from separate mice. Statistical analysis was performed using the unpaired Student's t-test, and a *p-*value less than 0.05 was considered statistically significant. A population pyramid from Stanford University was used to analyze patient data.

## SUPPLEMENTARY MATERIAL TABLE AND FIGURES



## Abbreviations

Yap1: Yes-associated protein 1
TICs: Tumor initiating cells
TPCs: Tumor propagating cells
NTCs: Non-tumorigenic cells
LDA: Limited dilution assay
RNA-seq: RNA sequencing
GFP: Green-fluorescence
ER: Estrogen receptor
ROS: Reactive oxygen species
TGF-β: Transforming growth factor β
mOS: median overall survival
