# Supplementary material for: Yap1 promotes the survival and self-renewal of breast tumor initiating cells via inhibiting Smad3 signaling
Source: Oncotarget. 2015 Dec 18;7(9):9692–706. doi: 10.18632/oncotarget.6655 (PMC4891077; doi:10.18632/oncotarget.6655)
Supplement: Supplementary file 1 [file oncotarget-07-09692-s001.pdf]

Yap1 promotes the survival and self-renewal of breast tumor initiating cells via inhibiting Smad3 signaling

Supplementary Material

Transgenic mice, tumor dissociation and FACS

MMTV-Wnt1 mice were purchased from the Jackson laboratory (Jackson lab, #002934) and used to generate the breast tumors adopted in this study. MMTV-Wnt1 murine breast tumors were harvested and dissociated into single cell suspensions, as previously described (1,2). Obtained cell suspensions were stained with the antibodies listed below, analyzed, and sorted with a FACS Aria II cell sorter (BD Biosciences). We used antibodies including anti-mouse CD49f-APC (1:200 dilution; eBiosence, #17-0495-82) and EpCAM-Pecy7 (1:200 dilution; Biolegend, #118206) for sorting TICs (CD49f^{high}EpCAM^{low}) and non-tumorigenic cells (NTCs, CD49f^{low}EpCAM^{high}). In all of the sorting experiments, CD45-PerCPcy5.5 (1:200 dilution; Biolegend, #103132) and CD31-PerCPcy5.5 (1:200 dilution; Biolegend, #102422) were used to indicate lineage negative (CD45^{-}CD31^{-}) epithelium and deplete non-epithelial cells. Cells were sorted into staining buffer, and a small sample of the sorted cells was examined for purity. Final cell purity was greater than 95%. All of the animal procedures were conducted following the animal regulations, guidelines and protocols that were approved by the Administrative Panel on Laboratory Animal Care (APLAC) at Stanford University.

Conditional Yap1 knock-out mice

We reconstructed an estrogen receptor 1 (ER)-induced conditional Yap1 knock-out mice model. A Yap1 flox mouse was kindly provided by Dr. Pan (3) and mated to Rosa26-Cre mice (Jackson lab, #012429), mTmG mice (Jackson lab, #007576) and MMTV-Wnt1 mice (Jackson lab, #002934) to generate MMTV-Wnt1/Rosa26-Cre/mTmG/Yap1$^{flox/flox}$ (Yap1-flp) mice. Like MMTV-Wnt1 mice, the Yap1-flp female mice gave rise to breast tumors. At Yap1$^{flox/flox}$ (Yap1-flp) background, the generated female mice not only had the MMTV-Wnt1 transgene but also the Rosa26-CreER2 transgene and reporter transgenic mTmG. The Rosa26-CreER2 transgene led to constitutive production of CreER2 protein, which could become active Cre recombinase upon binding to Tamoxifen. The mTmG reporter was used as an indicator of active Cre recombinase, which switched from red-fluorescence (tdTomato) to green-fluorescence (GFP) upon active Cre recombinase (Supplementary Figure S1A and S1B).

For the preparation of a Yap1 knock-out (Yap1-ko), Tamoxifen (Sigma, #T5648) was dissolved in 100% ethanol at a concentration of 5 mg/mL. Immediately before injection, 300 μl of corn oil (Sigma, C8267) was added to a 1.5 mL tube, and was then mixed with 1 mL of tamoxifen. Briefly, after vortexing and spinning down for a few seconds, a vacuum centrifuge was used to remove all of the ethanol so that tamoxifen could be mixed into the corn oil. Then, using a 1 mL syringe (BD, #309602) and a 21G 5/8 needle (BD, #305122), tamoxifen (50 mg/kg mouse) was injected

subcutaneously for 10 successive days to knock out the Yap1 gene *in vivo*. The injection was slow and the needle was left inside for approximately 2 seconds to minimize back flow or solution loss. After excitation with UV light, the organs of the Yap1-ko mouse were pink in contrast to those without tamoxifen injection. DNA extracting and genotyping from tail biopsies were performed as previously described (3). Primers for genotyping of Yap1 (P1, P2 and P3), Cre and Wnt (Supplementary Table S1) were used to identify wild-type Yap1 (control, 498 bp), Yap1 flox/flox (Yap1-flp, 597 bp), Yap1 knock-out (Yap1-ko, 697 bp), the Cre gene (421 bp), and the heterozygous Wnt gene (440 bp). A standard genotyping protocol followed the instruction of previous research (3) and that of the Jackson Laboratory.

Similarly, 1 μg/mL of 4-Hydroxytamoxifen (Sigma, #68047-06-3) was supplemented in culture medium to knock out the Yap1 gene *in vitro*. Alternatively, active adenovirus-Cre (Vector Biolabs, #1045), which carried the Cre recombinase gene was used to knock out the floxed-Yap1 allele (Yap1-flp) *in vitro*.

Western blotting

Nuclear and cytoplasmic proteins were extracted according to the manufacturer's instructions (Sigma, #NXTRACT). Western blotting was performed as described in detail previously (4). Briefly, 5 μg of cytoplasmic protein or nuclear protein lysate from sorted cells was denatured, electrophoresed on gradient SDS-PAGE gels, and transferred onto nitrocellulose membranes (Immobilon-P, Millipore, Bedford, MA). After blocking, primary antibodies were applied to the membranes followed by horseradish peroxidase-conjugated secondary antibody. The western blotting signals were visualized using ECL Chemiluminescence Reagent Plus (PerkinElmer Life Sciences, Boston, MA) and exposed to films. Exposed films were scanned, and the intensity of the signal was quantified using software Image J (NIH). Primary antibodies used in this study included anti-mouse Yap1 (Abcam, #ab56701), TAZ (Cell signaling, #4883), Histone H4 (Santa Cruz, #sc-25260), Hsp90 (Santa Cruz, #sc-8262), p-Smad2 (Santa Cruz, sc-101801), Smad3 (Cell signaling, #9513), p-Smad3 (Santa Cruz, #sc-11769), Krt14 (Santa Cruz, #sc-43310), Krt18 (Santa Cruz, #sc-45406) and p63 (Abcam, #ab124762).

Two-Dimensional (2D) tumor growth assay in collagen gel

All solutions were kept on ice during preparation. Collagen gel solution (Chemicon, #ECM675) was prepared according to the manufacturer's instructions (8 mL of collagen solution, 2 mL of 5x medium and 250 μL of neutralization solution). After mixing, 1.5 mL of the chilled collagen solution was added onto a 6-well tissue culture plate. The plate was transferred to a 37 ℃ incubator for 60 min to initiate polymerization of the collagen. After formation of the collagen gel, the collagen gel was covered with culture media containing cells with 3 mL of medium. The cells were incubated overnight or for several days at 37 ℃ with CO_2. The medium was changed daily. After 10-14 days, cells were stained by Giemsa solution (Sigma, #G9641) to observe the cell density and number. Regarding the cell

count in 2D culture, the cells were digested, resuspended and counted under an inverted light microscope.

Three-dimensional (3D) tumor spheroid assay in matrigel

FACS-sorted tumor cells were suspended in culture medium consisting of DMEM/F12 (Invitrogen), 20 ng/mL of mouse EGF (BD, #354001), 20 ng/mL of human FGF (BD, #354060), 10 μM of Y-27632 (Sigma, #Y0503) and 1x B27 (Invitrogen, #17504-044), and then plated on top of solidified matrigel (BD Bioscience, #356237). We diluted matrigel (5 mg/mL to 1 mg/mL) in serum free-cold cell culture media, put 100 μL of the diluted matrigel into the upper chamber of a 24-well transwell, and incubated the transwell in a 37 ℃ incubator for at least 1 h for gelling. Then, 100 μL of the cell suspension with 4k cells was put onto the matrigel. The lower chamber of the transwell was filled with 600 μL of culture media containing 5 μg/mL of fibronectin, as an adhesive substrate. Then, the 24-well plate was incubated at 37 ℃. The culture medium was replenished every 3 to 4 days. After 10-14 days, colonies bigger than 50 μm in diameter were counted under an inverted light microscope. To passage, the colonies were dissociated with 1 mg/mL of dispase (Invitrogen, #17105-041) and treated with Trypsin/0.05% EDTA (Gibco, #25200) to produce a single cell suspension (5). Cells were then resuspended in culture medium, counted, sorted and replated on top of matrigel.

References

1. Feng W, Gentles A, Nair RV, Huang M, Lin Y, Lee CY, Cai S, Scheeren FA, Kuo AH, Diehn M: Targeting Unique Metabolic Properties of Breast Tumor Initiating Cells. Stem Cells 2014, 32(7):1734-1745.
2. Cho RW, Wang X, Diehn M, Shedden K, Chen GY, Sherlock G, Gurney A, Lewicki J, Clarke MF: Isolation and molecular characterization of cancer stem cells in MMTV-Wnt-1 murine breast tumors. Stem Cells 2008, 26(2):364-371.
3. Zhang N, Bai H, David KK, Dong J, Zheng Y, Cai J, Giovannini M, Liu P, Anders RA, Pan D: The Merlin/NF2 tumor suppressor functions through the YAP oncoprotein to regulate tissue homeostasis in mammals. Dev Cell 2010, 19(1):27-38.
4. Huang M, Nguyen P, Jia F, Hu S, Gong Y, de Almeida PE, Wang L, Nag D, Kay MA, Giaccia AJ, Robbins RC, Wu JC: Double knockdown of prolyl hydroxylase and factor-inhibiting hypoxia-inducible factor with nonviral minicircle gene therapy enhances stem cell mobilization and angiogenesis after myocardial infarction. Circulation 2011, 124(11 Suppl):S46-54.
5. Lukacs RU, Goldstein AS, Lawson DA, Cheng D, Witte ON: Isolation, cultivation and characterization of adult murine prostate stem cells. Nat Protoc 2010, 5(4):702-713.

Table S1. Primers for qRT-PCR and genotype

Gene	Forward primer	Reverse primer
**qRT-PCR**		
β-actin	5’-tcactattggcaacgagcg-3’	5’-aggtctttacggatgtcaacg-3’
Yap1	5’-acgacttcctcaacagtgtg-3’	5’-tcattgcatctccttccagtg-3’
Krt14	5’-aggatgctgaggaatggttc-3’	5’-cgatctccaggttctgcatg-3’
p63	5’-gcattgtcagtttcttagcaagg-3’	5’-agatggcatgtcggaactg-3’
Krt18	5’-acaccaacatcacaaggctg-3’	5’-ttccacagtcaatccagagc-3’
Ecad	5’-agacgctgagcatgtgaag-3’	5’-tgtttcgaggttctgggatg-3’
Oct-4	5’-gaaggatgtggttcgagtatgg-3’	5’-actgtgtgtcccagtctttattt-3’
Sox-2	5’-agacgctcatgaagaaggataag-3’	5’-cctcccaattcccttgtatctc-3’
Nanog	5’-tgactccaccaggtgaaatatg-3’	5’-gtgagatggctcagtggataag-3’
Klf4	5’-acacaggcgagaaaccttac-3’	5’-catctgaaaccacagtgcataac-3’
Smad3	5’-ccgagaacactaacttccctg-3’	5’-catcttcactcaggtagccag-3’
**Genotype**		
Yap1	P1: 5’-ccatttgtcctcatctcttactaac-3’	P2: 5’-gattgggcactgtcaattaatgggctt-3’<br>P3: 5’-cagtctgtaacaaccagtcagggatac-3’
Cre	5’-gctggttagcaccgcaggtgtagag-3’	5’-cgccatcttccagcaggcgcacc-3’
Wnt hete	5’-ggacttgcttctcttctcatagcc-3’	5’-ccacacaggcatagagtgtctgc-3’
Wnt wt	5’-caaatgttgcttgtctggtg-3’	5’-gtcagtcgagtgcacagttt-3’

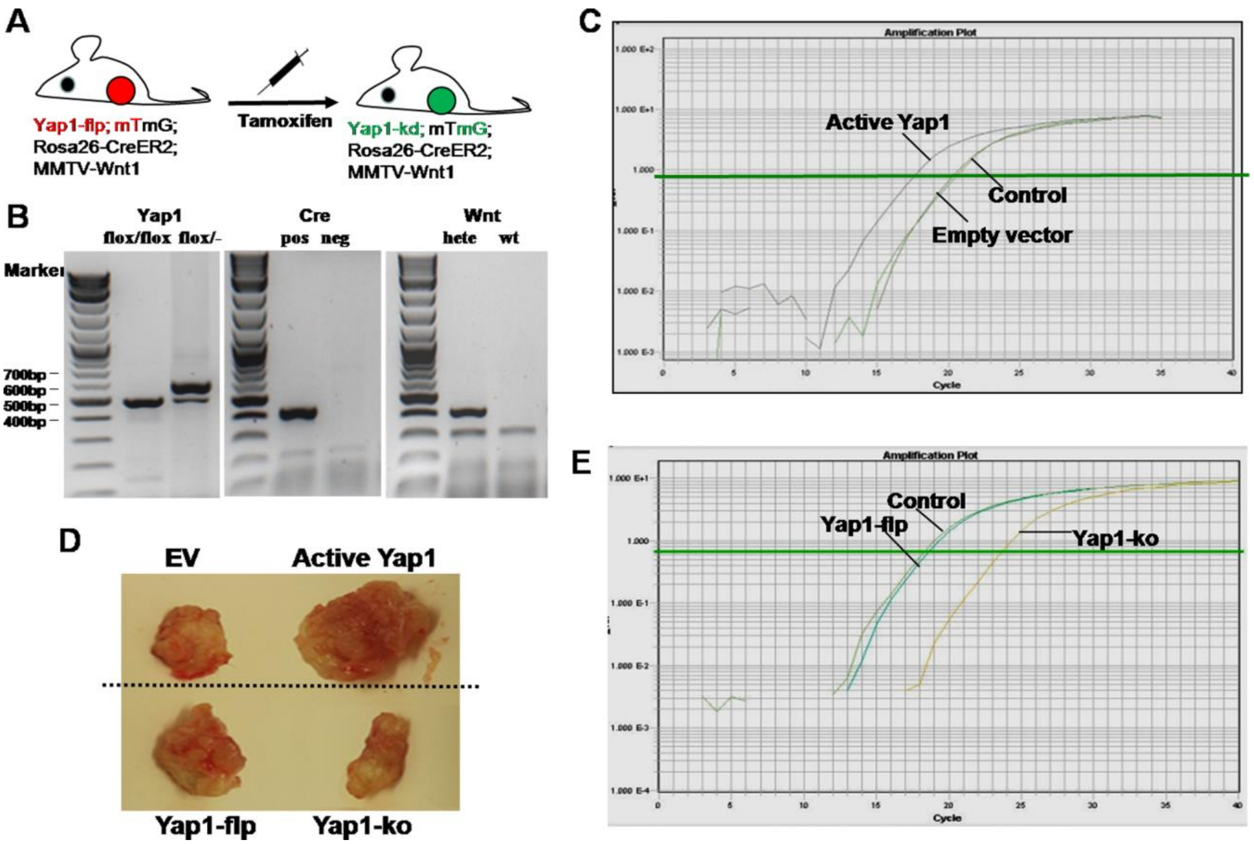

Figure S1. Yap1 activation was required for the expansion of breast tumor cells.

(A) Scheme for Yap1 deletion (knockout, KO) in MMTV-Wnt1 mammary tumors *in vivo*. Upon tamoxifen injection, active Cre recombinase switched the reporter gene mTmG from red (tdTomato) to green (GFP), and knockout Yap1 gene was flanked by loxP elements.

(B) The MMTV-Wnt1/Rosa26-Cre/mTmG/Yap1$^{flox/flox}$ (Yap1-flp) mice were obtained and confirmed by genotyping. Two genotype bands KO and Flp (two arrows) represented the Yap1 knockout allele and the intact Yap1 allele flanked that was by loxP sites, respectively.

(C) After lentivirus transfection, qRT-PCR showed more Yap1 in lentivirus-Yap1 transfected TICs than in parental TICs and empty vector (EV) transfected TICs.

(D) With the inoculation of the same number of TICs with or without active Yap1 in FVB/NJ female mice, active Yap1 formed dramatically bigger tumors than mice harboring empty vector transfected TICs.

(E) After Yap1-ko, qRT-PCR showed less Yap1 in lentivirus-Yap1 transfected TICs than in parental TICs and Yap1-flp transfected TICs.

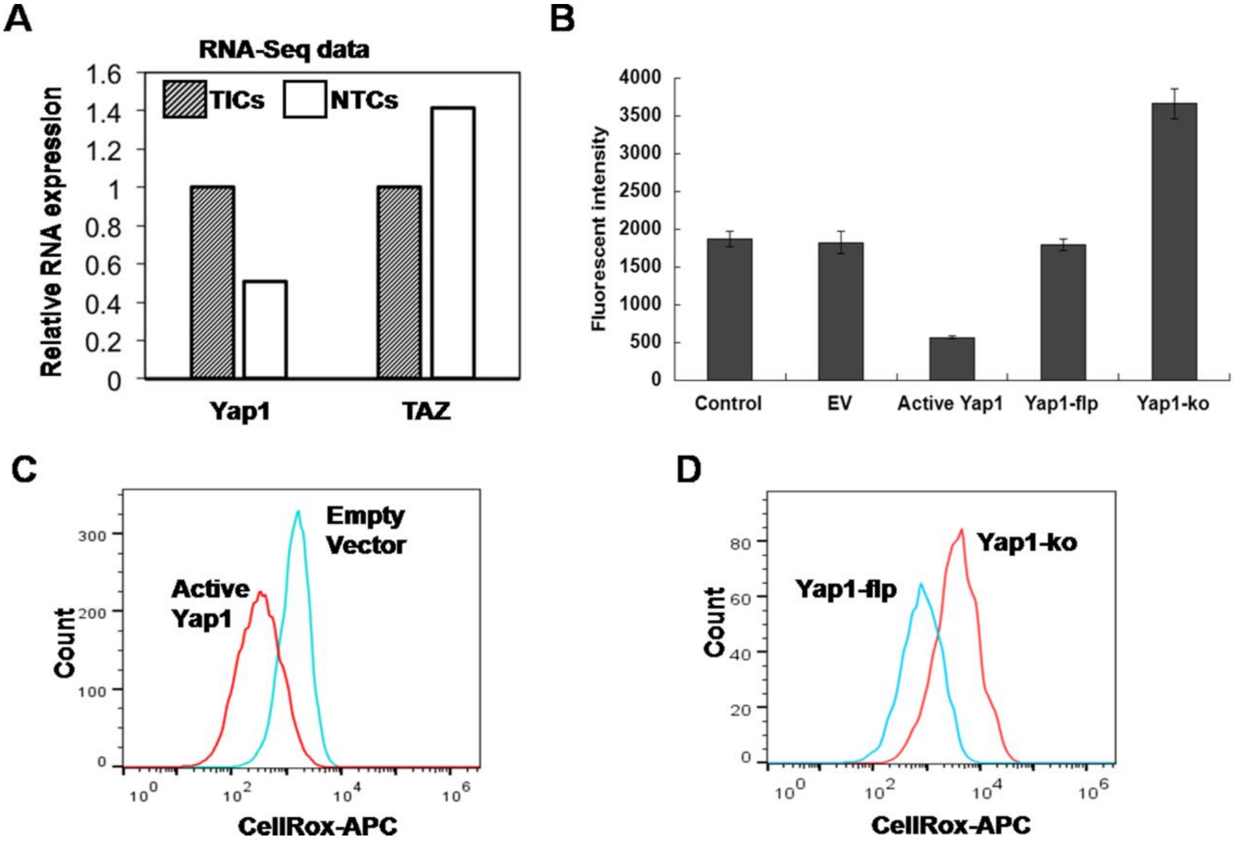

Figure S2. Active Yap1 decreased cytoplasmic ROS level in breast TICs.

(A) RNA-seq indicated that TAZ was lowly expressed in breast TICs, while Yap1 was specifically activated within these cells.

(B) Mean fluorescence intensity for CellRox with ectopic active Yap1 and Yap1-ko.

(C) Flow cytometry analysis showed ROS levels within mammary tumor cells with ectopic expression of active Yap1 using a CellRox assay.

(D) Flow cytometry analysis showed ROS levels within mammary tumor cells with ectopic expression of Yap1 knockout (KO) using a CellRox assay.
